# Giant deep orbital dermoid cyst presenting early in infancy in a Nigerian child: a case report and review of the literature

**DOI:** 10.1186/1752-1947-6-320

**Published:** 2012-09-25

**Authors:** Oluyemi Fasina, Olabiyi G Ogun

**Affiliations:** 1 Department of Ophthalmology, University College Hospital Ibadan, Ibadan, Nigeria; 2 Department of Pathology, University College Hospital Ibadan, Ibadan, Nigeria

## Abstract

**Introduction:**

Dermoid cysts are the most common orbital cystic lesions seen in children. While superficial orbital dermoid cysts present early in life, deep dermoid cysts remain clinically occult until adolescence or adulthood. We here present a case of a deep orbital dermoid cyst in a Nigerian child that became symptomatic early in infancy.

**Case presentation:**

A female Nigerian infant of Yoruba ethnicity presented at three months of age with left non-axial proptosis and a hazy cornea. A superotemporal cystic orbital mass was seen on ultrasonography, and her parents were counseled for simple tumor excision. They however defaulted, only for their child to re-present two years later with gradually progressive proptosis, an enlarged orbit and keratinized ocular surface, necessitating orbital exenteration.

**Conclusion:**

Deep orbital dermoid cysts may be symptomatic from birth. Late presentation may result in an irreversible loss of vision, as demonstrated in our case. The need for public enlightenment on early presentation and prompt management of such benign lesions is emphasized.

## Introduction

Dermoid cysts are congenital, benign, cystic teratomas [1], described as developmental cystic lesions derived from inclusion of ectodermal elements during the closure of the neural tube adjacent to fetal suture lines [2]. They are choristomas, tumors that emanate from aberrant primordial tissue, and consist of normal appearing tissues in an abnormal location. They are by far the most common orbital cystic lesions encountered in children, accounting for 3% to 9% of all orbital tumors, with an average of 4.7% [3]. In a series by Sherman *et al*. [4], they comprise 6% of the orbital tumors reviewed. Incidence varies from as low as 1.6% to as high as 46% [5] in different studies. Studies reporting both clinically diagnosed and biopsied cases have lesser incidence compared with those reporting only biopsied cases [2]. Reported incidence from previous studies in Nigeria ranges from 1.6% to 4.5% [6,7].

Dermoid cysts are usually classified as juxtasutural, sutural or of soft tissue types, with further subdivisions, based on their relationship to the orbital bone and location within the soft tissues [8]. However, they can generally be divided into either superficial (simple, exophytic) or deep (complicated, endophytic) dermoid cysts based on their relationship to the orbital septum [4,9]. Superficial dermoid cysts usually present early in life as a slowly growing mass, sometimes discovered accidentally by the child’s parents [2]. They are rarely painful, and the eyelid and ocular functions are not affected [4]. Deep orbital dermoid cysts generally remain clinically occult until adolescence or adulthood, when they enlarge and cause proptosis [1,2,10].

On histological examination, these tumors are seen as keratin-containing cavities surrounded by stratified squamous epithelial walls with skin appendages such as sebaceous glands and hair follicles.

Dermoid cysts are generally benign noninvasive lesions that rarely cause orbital damage. However, a review by Bonavolonta and associates [9] found that 14% of the lesions caused considerable destruction of adjacent bony structures. Occasionally, the lesions can extend through the roof of the orbit into the frontal sinus [1]; rupture spontaneously, inciting intense orbital inflammatory response [8]; or drain intermittently though a secondary fistula to the skin [11].

## Case presentation

A three-month old female baby of Yoruba ethnicity presented to our Eye Clinic with gradually progressive left proptosis and a white spot noticed in her left eye from birth. She was the product of full-term pregnancy and normal vaginal delivery. She had no significant family history and an examination revealed an infant in good general health condition. She had left non-axial proptosis; a tense orbit; keratinized, hazy cornea; and inadequate lid closure. There were no masses palpable in her orbit. Ultrasonography revealed a 23mm superotemporal cystic mass with membranous speckled content in her left orbit, with a 9mm retro-ocular extension. Her left globe was distorted and irregular, but no intra-ocular mass was seen. Ocular axial length measurement with an amplitude modulation scan and computed tomography were not done due to financial constraints. She was scheduled for a detailed examination including intra-ocular pressure measurement, exophthalmometry and simple tumor excision under general anesthesia. The parents, however, defaulted.

**Figure 1:**
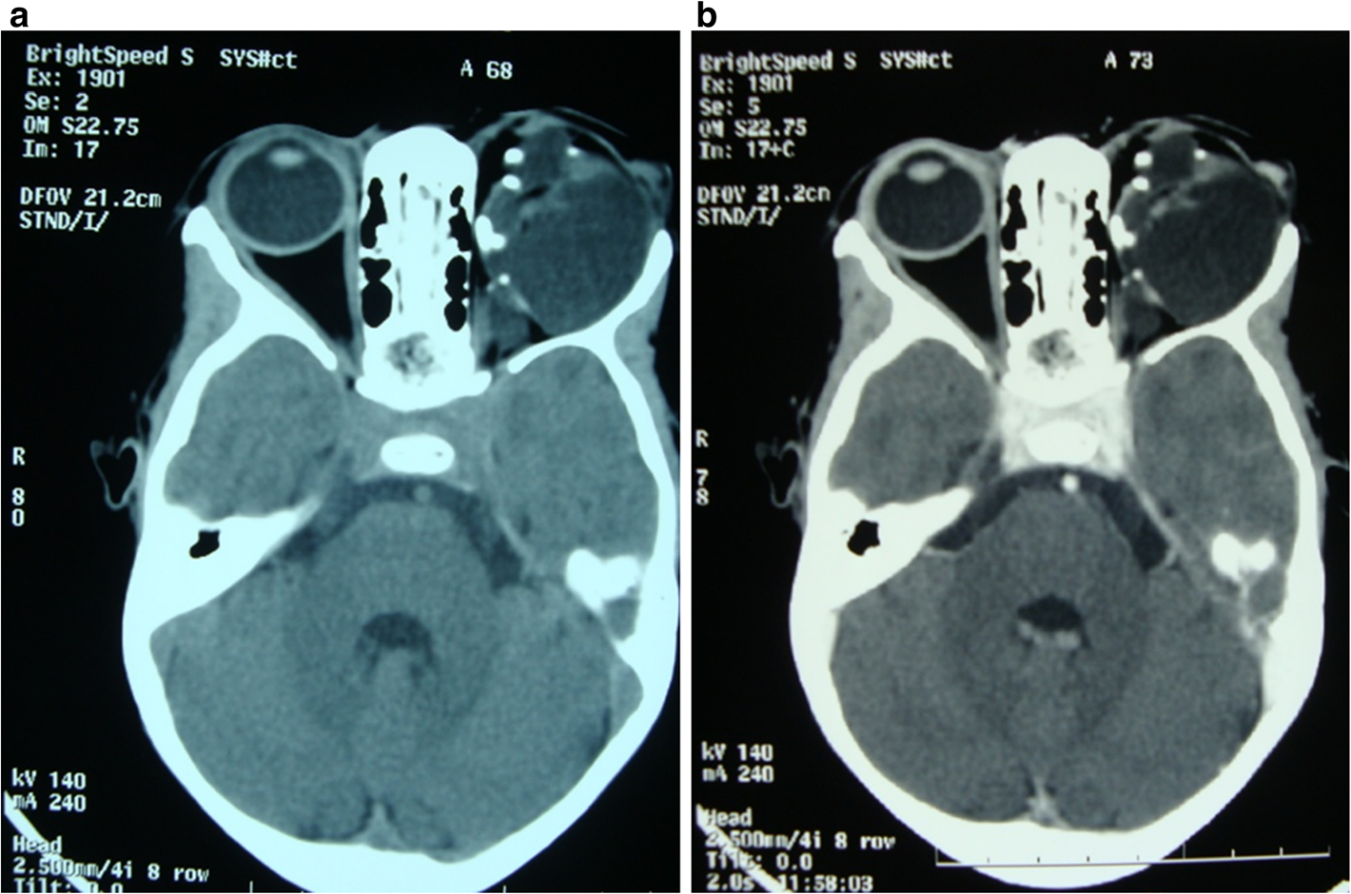
**Computed tomographic scan.** (**a**) This shows a huge, non-enhancing mixed density mass with areas of calcification. (**b**) The orbit is enlarged with thinned wall.

Our patient re-presented two years later with continued slowly progressive non-axial proptosis, worsening inadequate lid closure, corneal opacity and a keratinized ocular surface. A computed tomographic scan done at this time showed a huge, non-enhancing mixed density mass with areas of calcification. Her orbit was enlarged, with thinned walls, but no bone destruction was seen (Figure 1a,b). She subsequently underwent lid-sparing orbital exenteration, with dissection up to the periosteum, thereby preventing disruption of the sac of the cyst.

**Figure 2:**
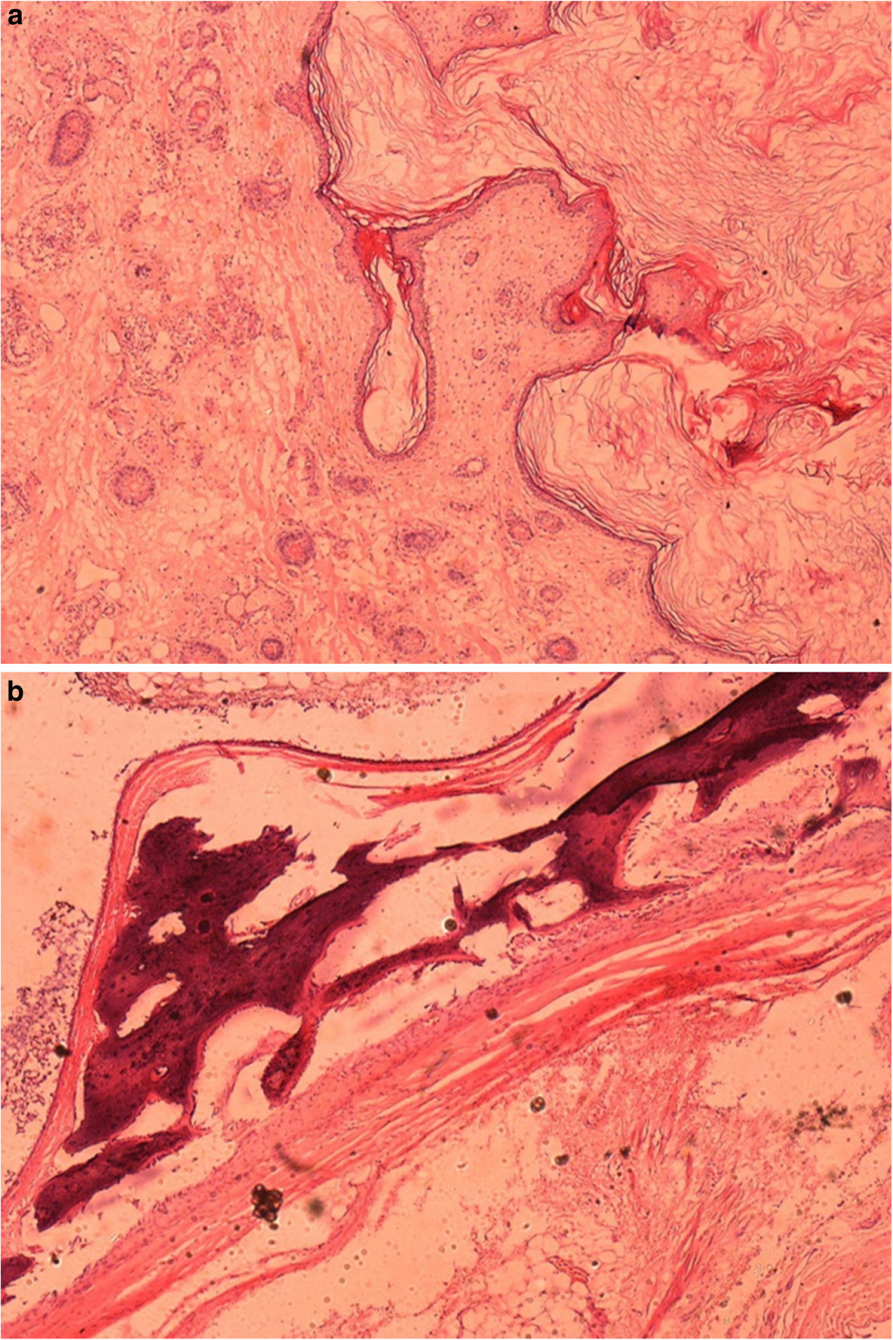
**Photomicrograph of the histology slide (hematoxylin and eosin staining).** (**a**) The cyst was lined by keratinized stratified squamous epithelium with keratin material in the lumen. (**b**) Dystrophic calcification and adipocytic cells in the cyst wall.

On gross pathologic examination her eyeball was surrounded by fibroadipose tissue and an extra-ocular tumor located posteriorly, measuring about 3×2×2cm in size. A cut section of the tumor revealed a cystic cavity containing hair shaft admixed with thick yellowish material and firm to hard tissue. Microscopic examination showed a cyst containing keratin material in the lumen, lined by keratinized stratified squamous epithelium. Sebaceous and sweat glands and hair follicles were associated with the wall (Figure 2a). The cell wall also had dystrophic calcification with ossification and included adipocytic cells (Figure 2b).

## Discussion

Epidermal dermoid cysts are believed to develop from epidermal rest cells that get entrapped in the deeper tissues during embryonic development. This explains their tendency to occur in the orbit at sites of bony fusion, particularly along the zygomaticofrontal suture [1]. Their natural history is of a gradually enlarging cystic mass with displacement of adjacent structures, although some may remain relatively dormant [4,11]. Spontaneous or traumatic rupture of the cyst occasionally occurs, resulting in inflammation and a rapid increase in the size of the lesion.

Microscopic examination of such lesions reveals chronic granulomatous inflammation in the epithelial wall [8]. These epithelioid and foreign body giant cells were, however, absent in our case. Thus, small asymptomatic orbital dermoid cysts may not require any immediate treatment, as they can remain stable for years or reduce in size [1]. However, deeper seated lesions frequently present later in life as giant dermoid cysts that require excision, and may present a surgical challenge [2,4,8]. Similar to previous reports [8,12], the cystic mass was located in the superotemporal quadrant portion of the orbit, but with extension into the posterior orbit in our patient. Sherman and associates [4] reported on five patients with deep orbital dermoid cysts, aged 15 to 40 years. All presented with normal visual acuity and ocular motility and subsequently had simple tumor excision. A similar case was described by Bickler-Bluth *et al*. [13]. One of the patients in the series by Shields and associates [8] presented with a giant orbital cyst at birth, similar to our case, but with well preserved ocular anatomy. Early presentation and surgical intervention has been advocated as the key to a good outcome in this group of patients [10].

Late presentation has affected the management of many benign ophthalmic conditions in developing countries, and it is not unusual for the parents to have tried other remedies before finally presenting to the hospital or agreeing with the treatment offered [14]. Superstitious beliefs and financial constraints [15] were identified as reasons for patients in our environment presenting late to the eye-care facilities, with consequent poor outcome in management. Our patient initially presented at three months of age when the cornea was already hazy, subsequently defaulted from the surgical treatment offered, and re-presented after two years with large proptosis, a massively expanded orbit, lagophthalmos, and a keratinized ocular surface from exposure keratopathy. At this time, the best surgical intervention we could offer was lid-sparing exenteration. Simple tumor excision could have been done if the parents had presented our patient earlier, sparing the globe and, possibly, providing a good visual prognosis.

## Conclusion

Previously reported dermoid cysts in our environment [6,7] formed part of a series of orbito-ocular lesions and were superficial. We present an unusual case of a deep orbital dermoid cyst in a child.

There is an urgent need to educate the medical staff and general populace on these benign childhood orbital conditions and their uncommon presentation. Parents should be encouraged to present their children early to health facilities for treatment of this benign condition. This could have led to the preservation of visual function in our patient, and may preserve life in other potentially life-threatening conditions.

## Consent

Written informed consent was obtained from the patient’s father for publication of this case report and accompanying images. A copy of the written consent is available for review by the Editor-in-Chief of this journal.

## Competing interests

The authors declare that they have no competing interests.

## Authors’ contributions

OF analyzed and interpreted the patient’s clinical data regarding the ophthalmic condition. OGO performed the histopathological examination of the specimen, and was a contributor in writing the manuscript. Both authors read and approved the final manuscript.
